# RAGE inhibition reduces acute lung injury in mice

**DOI:** 10.1038/s41598-017-07638-2

**Published:** 2017-08-03

**Authors:** Raiko Blondonnet, Jules Audard, Corinne Belville, Gael Clairefond, Jean Lutz, Damien Bouvier, Laurence Roszyk, Christelle Gross, Marilyne Lavergne, Marianne Fournet, Loic Blanchon, Caroline Vachias, Christelle Damon-Soubeyrand, Vincent Sapin, Jean-Michel Constantin, Matthieu Jabaudon

**Affiliations:** 1 Department of Perioperative Medicine, CHU Clermont-Ferrand, Clermont-Ferrand, France; 2 Université Clermont Auvergne, CNRS, INSERM, GReD, F-63000 Clermont–Ferrand, France; 3 Department of Medical Biochemistry and Molecular Biology, CHU Clermont-Ferrand, Clermont-Ferrand, France

## Abstract

The receptor for advanced glycation end-products (RAGE) is involved in inflammatory response during acute respiratory distress syndrome (ARDS). Growing body of evidence support strategies of RAGE inhibition in experimental lung injury, but its modalities and effects remain underinvestigated. Anesthetised C57BL/6JRj mice were divided in four groups; three of them underwent orotracheal instillation of acid and were treated with anti-RAGE monoclonal antibody (mAb) or recombinant soluble RAGE (sRAGE), acting as a decoy receptor. The fourth group served as a control. Lung injury was assessed by the analysis of blood gases, alveolar permeability, histology, AFC, and cytokines. Lung expression and distribution epithelial channels ENaC, Na,K-ATPase, and aquaporin (AQP)−5 were assessed. Treatment with either anti-RAGE mAb or sRAGE improved lung injury, arterial oxygenation and decreased alveolar inflammation in acid-injured animals. Anti-RAGE therapies were associated with restored AFC and increased lung expression of AQP-5 in alveolar cell. Blocking RAGE had potential therapeutic effects in a translational mouse model of ARDS, possibly through a decrease in alveolar type 1 epithelial cell injury as shown by restored AFC and lung AQP-5 expression. Further mechanistic studies are warranted to describe intracellular pathways that may control such effects of RAGE on lung epithelial injury and repair.

## Introduction

Acute respiratory distress syndrome (ARDS) is a syndrome of diffuse inflammatory lung injury with increased pulmonary oedema and the rapid onset of hypoxemic respiratory failure^1^. ARDS is still undertreated^2^, with high mortality and few effective therapies^3–5^. RAGE is a membrane receptor that is expressed in alveolar type (AT)-1 epithelial cells of the lung and a marker of epithelial injury^6^. There are many RAGE ligands, including high-mobility group box 1 protein (HMGB1), advanced glycation end-products (AGEs) and S100 protein^7, 8^. RAGE controls a variety of cellular processes such as cell proliferation and migration, inflammation, apoptosis and microtubule stabilization^9^. Its main soluble forms, referred to as soluble RAGE (sRAGE), include the extracellular domain of membrane RAGE (mRAGE) which is cleaved by proteinases and endogenous secretory RAGE (esRAGE, produced after alternative splicing)^10^. In clinical ARDS, sRAGE has good diagnostic value and is associated with lung injury severity, impaired alveolar fluid clearance (AFC) and prognosis^6, 11–13^.

Impaired AFC is a major feature of ARDS that contributes to mortality^14^. The main mechanism responsible for the resolution of alveolar oedema is ion transport across the alveolar epithelium, primarily through epithelial sodium (ENaC), Na,K-ATPase and aquaporin (AQP)-5 channels, thus creating a local osmotic gradient to reabsorb the water fraction of the oedema fluid from the airspaces of the lungs^15–17^. Recent data support an effect of RAGE activation on ENaC activity in cultured AT-1 cells^18^. However, in contrast to the situation in mice, the clearance of alveolar fluid after birth in humans may not critically depend on ENaC, at least in part because of greater reliance on other epithelial channels^15^. The modulation of RAGE may reduce inflammatory responses in several models^19^. Intratracheal administration of HMGB1 induced lung injury in mice and the pathological effects of intratracheal lipopolysaccharide (LPS) were partially ameliorated by systemic administration of anti-HMGB1 antibodies^8^, thereby implicating pattern-recognition receptors such as RAGE or toll-like receptors in the pathogenesis of ARDS. Experimental murine pulmonary ischemia followed by reperfusion caused lung injury that was ameliorated in mice treated with sRAGE and in RAGE^−/−^ mice^20^. Using a mouse model of lung injury induced by intratracheal LPS, Zhang *et al*. reported that sRAGE, a decoy receptor that prevents the interaction of RAGE with its ligands, significantly attenuated the increases in neutrophil infiltration, lung permeability, production of inflammatory cytokines, NF-κB activation, and apoptotic cells in the lung^21^. In rabbits, injurious high-tidal volume ventilation caused lung injury, with increased HMGB1 content in bronchoalveolar lavage (BAL) when compared with lower tidal volumes, an effect attenuated by anti-HMGB1 antibodies^22^. Whereas RAGE was not described as a prominent contributor to the pro-inflammatory state induced by injurious ventilation in mice^23^, treatment with sRAGE limited the production of pro-inflammatory mediators in a two-hit model of ARDS caused by LPS plus high-tidal volume ventilation. Modulation of RAGE expression or activity could therefore reduce pro-inflammatory processes in other experimental models of nonpulmonary sepsis. Indeed, the use of recombinant sRAGE decreases inflammatory responses and improves survival in a model of peritonitis^24^. In addition, anti-RAGE monoclonal antibody (mAb) has protective effects in mice with sepsis^25^.

Because modalities of RAGE inhibition vary among published studies, and because its modalities may directly impact its effects in a given model, this study was designed to investigate the influence of a treatment with either sRAGE or anti-RAGE mAb on the features of experimental ARDS, including AFC, and on lung expression of alveolar epithelial channels in a translational model of acid-induced lung injury^13, 26^.

## Results

### Physiological dysfunction

**Figure 1:**
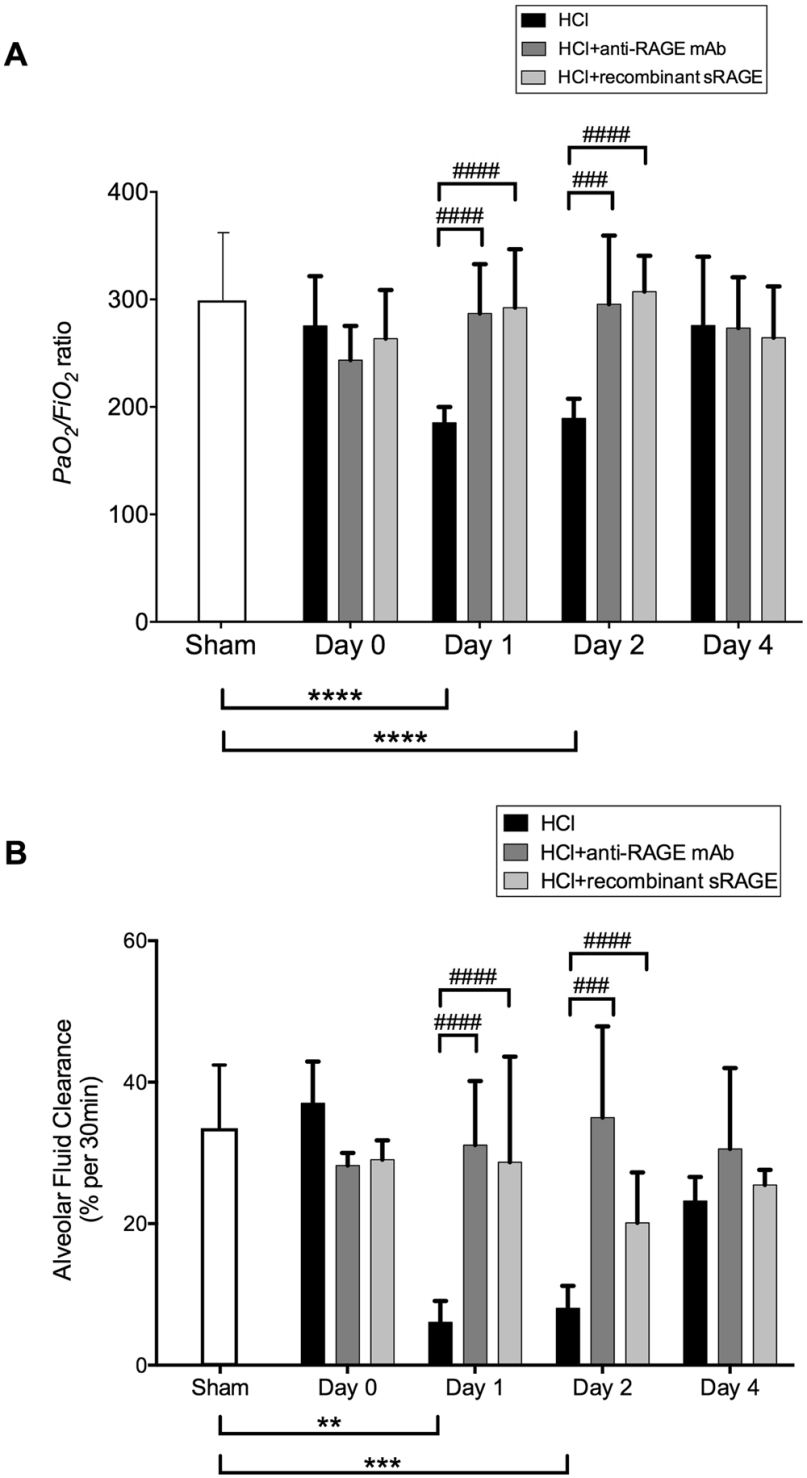
RAGE inhibition improves arterial oxygenation and alveolar fluid clearance. **(A)** Arterial oxygen tension (*PaO*_*2*_)/inspiratory oxygen fraction (*FiO*_*2*_) in uninjured, untreated mice (Sham group), acid-injured animals (HCl group), acid-injured animals treated with sRAGE (HCl + recombinant sRAGE group) or with anti-RAGE monoclonal antibody (HCl + anti-RAGE mAb group)(n = 4–6 for each time-point). **(B)** Measurement of net alveolar fluid clearance (AFC) rate as a marker of epithelial function in acid-injured animals (HCl), acid-injured animals treated with sRAGE (HCl + recombinant sRAGE) or with anti-RAGE mAb (HCl + anti-RAGE mAb) (n = 4–6 for each time-point). As no difference was observed between sham animals at all time points; the results from sham mice were mixed for analyses (left bar of the X-axis). Values are reported as means ± standard deviations. **P < 10^−2^; ***P < 10^−3^; ****P < 10^−4^ versus sham controls; ^###^P < 10^−3^; ^####^P < 10^−4^ versus acid-injured animals.

In acid-injured mice, arterial oxygenation had deteriorated one day after injury, as compared with sham animals, with gradual improvement by day 4 (Fig. 1A). Mean arterial oxygen tension (*PaO*_*2*_ */FiO*_*2*_ ratios) therefore met clinical ARDS criteria on days 1–2 in injured mice, but not in injured mice treated with anti-RAGE mAb or sRAGE, in which *PaO*_*2*_ */FiO*_*2*_ were similar to those seen in sham animals. Net AFC rate was significantly impaired, as compared with sham animals, during the first 2 days after injury in HCl-treated animals, with the lungs regaining the ability to clear fluid on day 4. In contrast, RAGE inhibition restored AFC in acid-induced mice (Fig. 1B).

### Alteration of the alveolar capillary barrier

**Figure 2:**
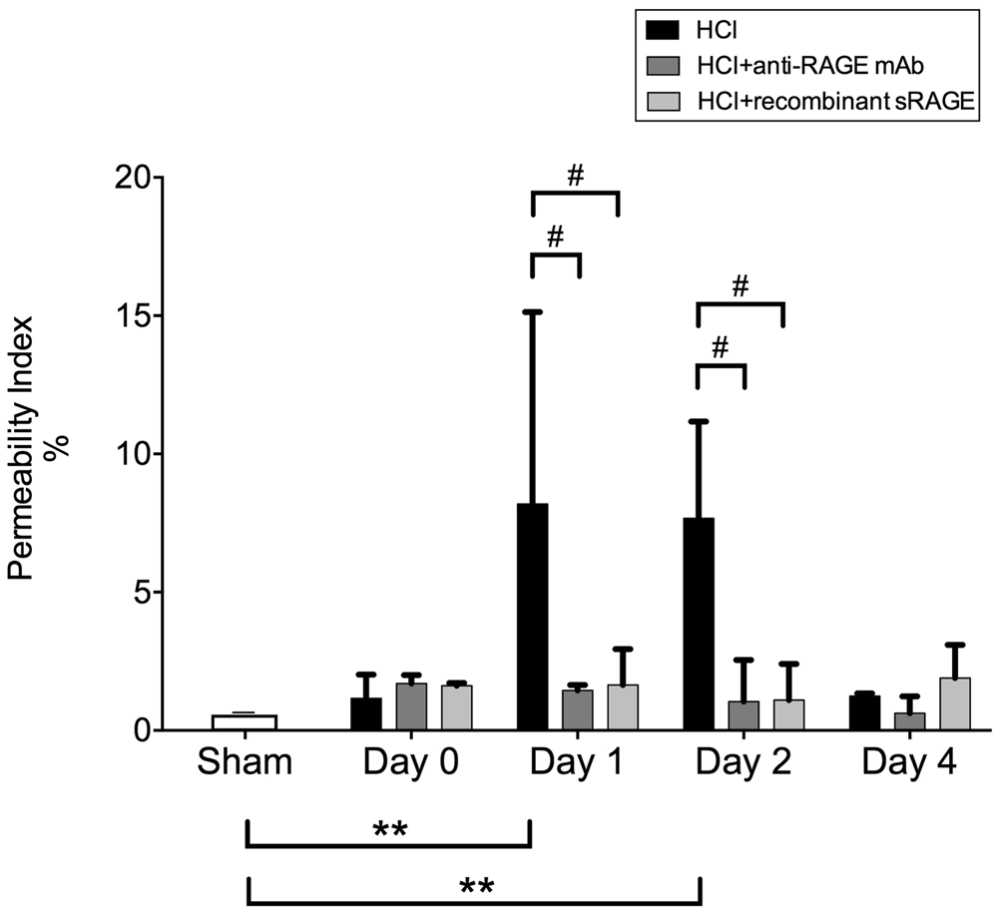
RAGE inhibition enhances the alveolar capillary barrier. The permeability index calculated as the bronchoalveolar lavage (BAL) fluid-to-plasma ratio of the human serum albumin (HSA) concentration in uninjured, untreated mice (Sham group), acid-injured animals (HCl group) and acid-injured animals treated with sRAGE (HCl + recombinant sRAGE group) or with anti-RAGE monoclonal antibody (HCl + anti-RAGE mAb group) (n = 4–6 for each time-point). As no difference was observed between sham animals at all time points; the results from sham mice were mixed for analyses (left bar of the X-axis). Values are reported as means ± standard deviations. **P < 10^−2^ versus sham controls, ^#^P < 0.05 versus acid-injured animals.

Alveolar-capillary barrier permeability, as assessed by the permeability index, showed a substantial increase on days 1–2 (with a return to normal levels by day 4) in injured animals, as compared with injured mice on day 0 and with sham animals at the same time-points. Treatment with either sRAGE or anti-RAGE mAb was efficient in normalising the permeability index on days 1 and 2 after acid injury (Fig. 2). BAL levels of total proteins were increased on day 1 in acid-injured mice, compared to sham animals and animals treated with anti-RAGE therapies (Supplement, Fig. S1).

### Lung epithelial injury

**Figure 3:**
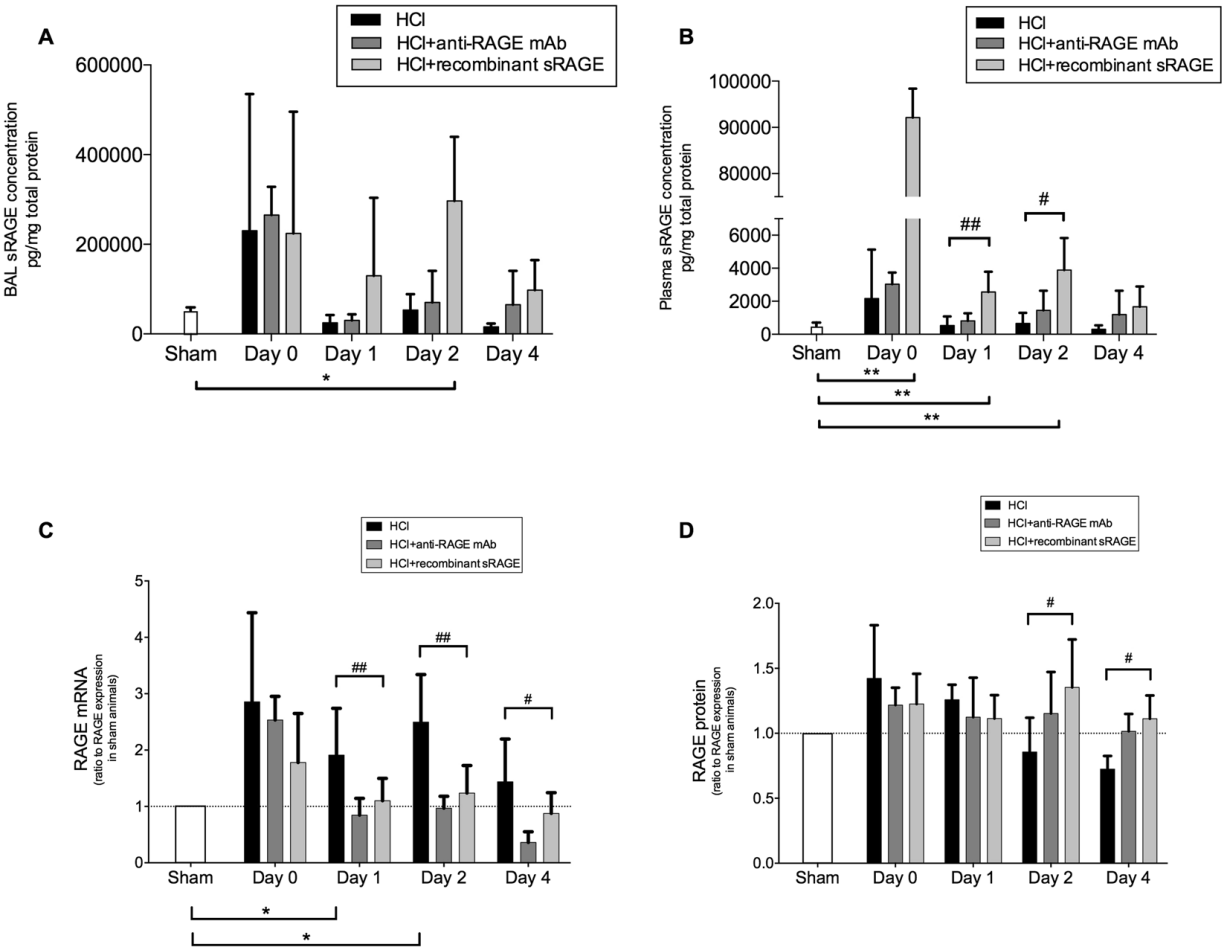
RAGE inhibition decreases lung epithelial injury. Levels of **(A)** bronchoalveolar fluid lavage (BAL), **(B)** plasma sRAGE, **(C)** lung RAGE mRNA, and **(D)** protein expression in the uninjured, untreated mice (Sham group), acid-injured animals (HCl group), acid-injured animals treated with sRAGE (HCl + recombinant sRAGE group) or with anti-RAGE monoclonal antibody (HCl + anti-RAGE mAb group) (n = 4–6 for each time-point). Levels of sRAGE and RAGE proteins were measured in duplicate *via* ELISA. Levels of BAL and plasma sRAGE were normalised to those of total protein. Levels of mRNA expression (∆∆Ct) were normalised to housekeeping genes. Protein and mRNA expression levels are expressed as ratios to those in sham controls. As no difference was observed between sham animals at all time points; the results from sham mice were mixed for analyses (left bar of the X-axis). Values are reported as means ± standard deviations. *P < 0.05; **P < 10^−2^ versus sham controls; ^#^P < 0.05, ^##^P < 10^−2^ versus acid-injured animals.

Acid-induced lung injury increased BAL and plasma levels of sRAGE on day 1; then, sRAGE levels decreased near baseline. Mice treated with sRAGE had higher levels of sRAGE in both BAL and plasma over time (Fig. 3A–B). Acid-induced mice had upregulated lung RAGE mRNA expression, whereas treatment with sRAGE or anti-RAGE mAb decreased lung RAGE mRNA levels on days 1–4 (Fig. 3C). In contrast, RAGE protein expression in the lung was not modified on day 1 after injury, but decreased on days 2–4 compared to sham animals. Anti-RAGE therapy restored lung RAGE protein expression on days 2–4, compared to untreated animals (Fig. 3D).

### Inflammatory response

**Figure 4:**
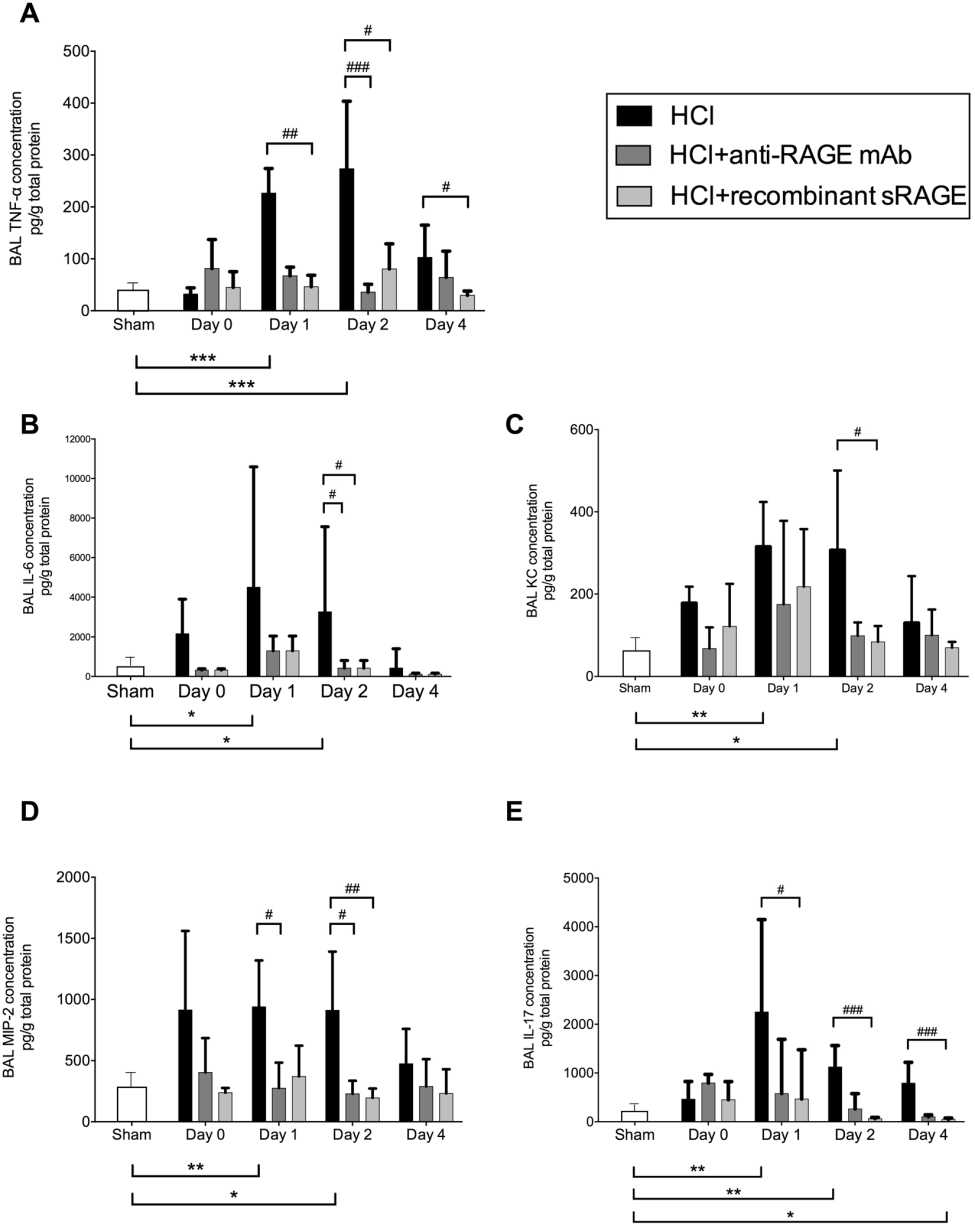
Anti-inflammatory effects induced by RAGE inhibition. Measurement of bronchoalveolar (BAL) levels of **(A)** interleukin (IL)-6, **(B)** tumor necrosis factor (TNF)-α, **(C)** keratinocyte-derived chemokine (KC), **(D)** macrophage inflammatory protein (MIP)-2 and **(E)** interleukin (IL)-17 in uninjured, untreated mice (Sham group), acid-injured animals (HCl group) and acid-injured animals treated with sRAGE (HCl + recombinant sRAGE group) or with anti-RAGE monoclonal antibody (HCl + anti-RAGE mAb group) (n = 4–6 for each time-point). Levels of cytokines were normalised to those of total protein. As no difference was observed between sham animals at all time points; the results from sham mice were mixed for analyses (left bar of the X-axis). Values are reported as means ± standard deviations. *P < 0.05; **P < 10^−2^; ***P < 10^−3^ versus sham controls; ^#^P < 0.05; ^##^P < 10^−2^; ^###^P < 10^−3^ versus acid-injured animals.

**Figure 5:**
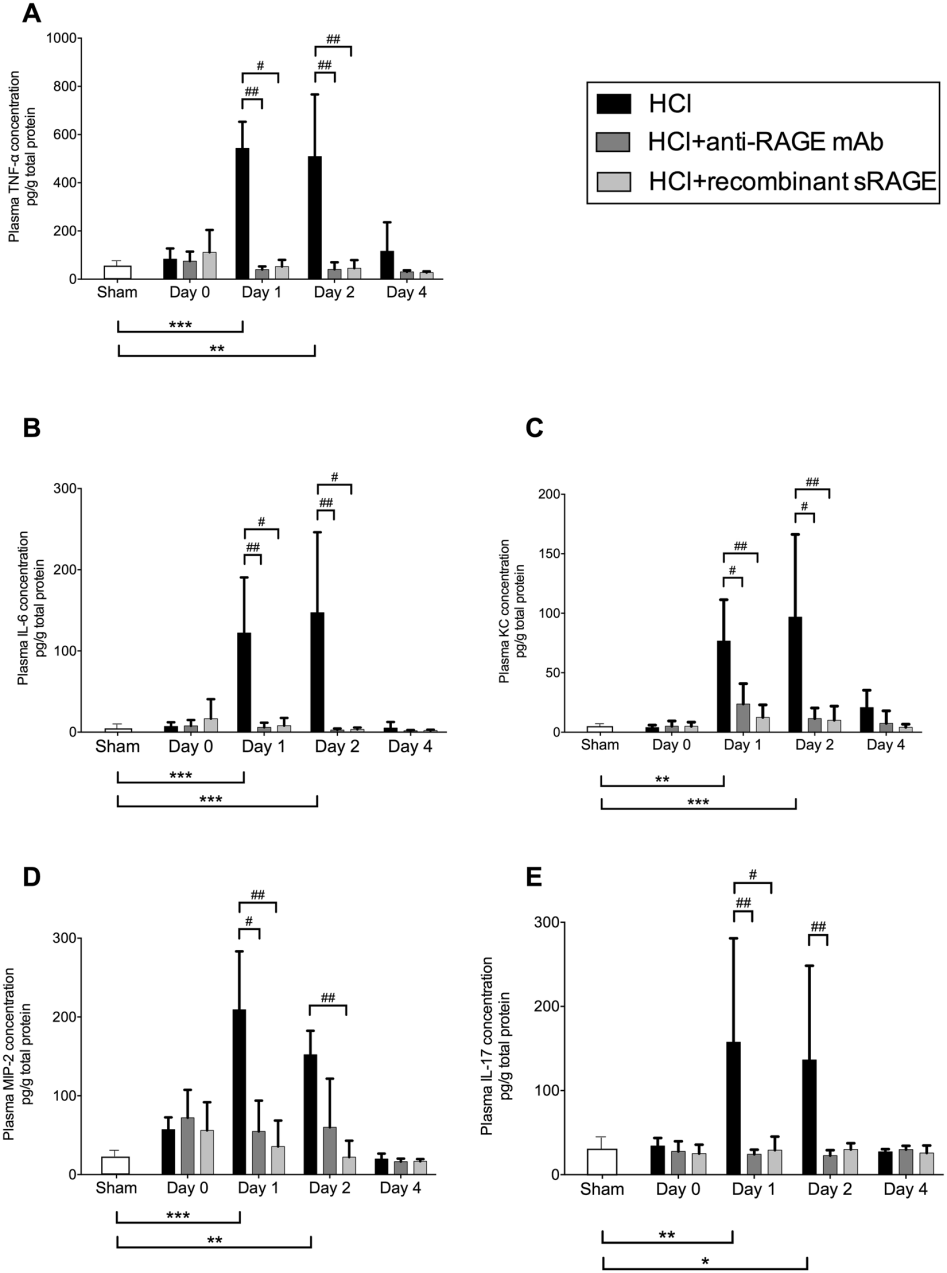
Anti-inflammatory effects induced by RAGE inhibition. Measurement of plasma levels of **(A)** TNF-α, **(B)** IL-6, **(C)** KC, **(D)** MIP-2 and **(E)** IL-17 in uninjured, untreated mice (Sham group), acid-injured animals (HCl group) and acid-injured animals treated with sRAGE (HCl + recombinant sRAGE group) or with anti-RAGE monoclonal antibody (HCl + anti-RAGE mAb group) (n = 4–6 for each time-point). Levels of cytokines were normalised to those of total protein. As no difference was observed between sham animals at all time points; the results from sham mice were mixed for analyses (left bar of the X-axis). Values are reported as means ± standard deviations. *P < 0.05; **P < 10^−2^; ***P < 10^−3^ versus sham controls; ^#^P < 0.05; ^##^P < 10^−2^ versus acid-injured animals.

**Figure 6:**
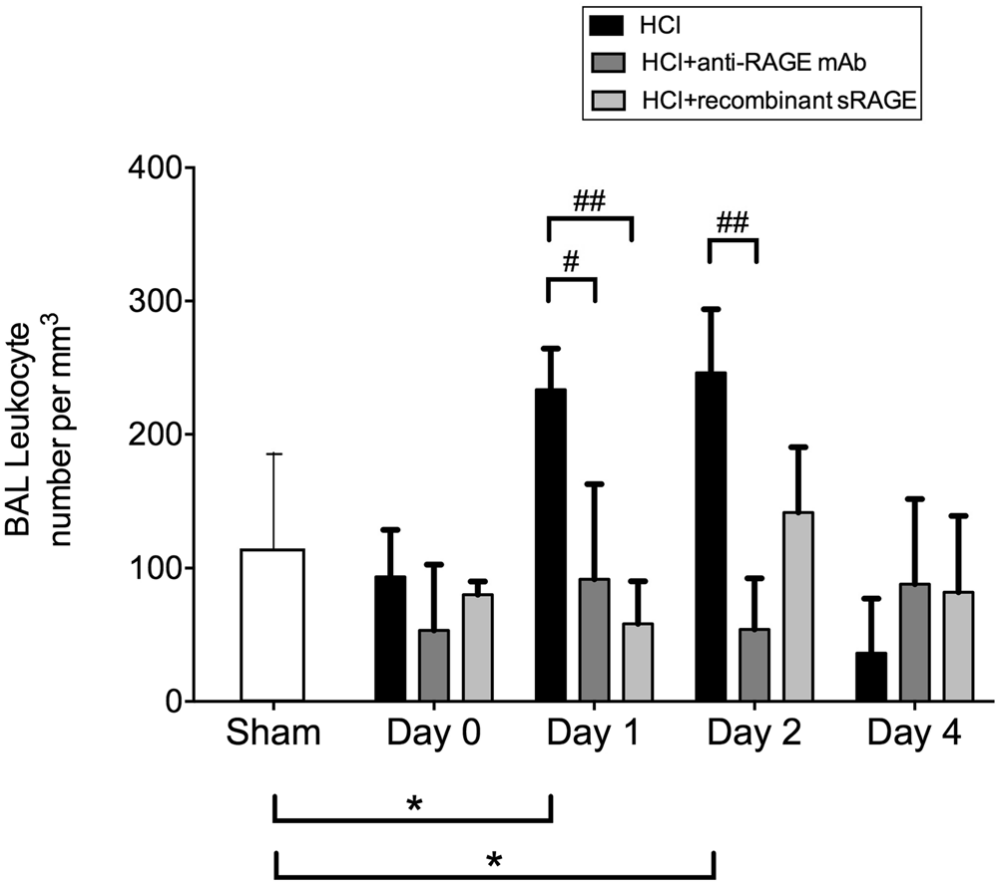
RAGE inhibition decreases the absolute number of leucocytes in the bronchoalveolar lavage (BAL). Total numbers of BAL leucocytes in uninjured, untreated mice (Sham group), acid-injured animals (HCl group) and acid-injured animals treated with sRAGE (HCl + recombinant sRAGE group) or with anti-RAGE monoclonal antibody (HCl + anti-RAGE mAb group) (n = 4–6 for each time-point). As no difference was observed between sham animals at all time points; the results from sham mice were mixed for analyses (left bar of the X-axis). Values are reported as means ± standard deviations. *P < 0.05 versus sham controls; ^#^P < 0.05; ^##^P < 10^−2^ versus acid-injured animals.

Following acid instillation, cytokine and chemokine levels were increased in BAL (Fig. 4) and plasma (Fig. 5) as compared to sham animals. Mice treated with anti-RAGE mAb or sRAGE had lower alveolar and plasma IL-6, TNF-α, KC, MIP-2 and IL-17 on days 1–2. On day 4, only the BAL TNF-α and IL-17 remained higher in injured mice than in sham animals, an effect significantly attenuated by treatment with sRAGE or anti-RAGE mAb. The number of total leukocytes in the BAL fluid was increased on days 1–2 after injury, and this phenomenon was significantly attenuated by anti-RAGE therapy (Fig. 6).

### Histological evidence of tissue injury

**Figure 7:**
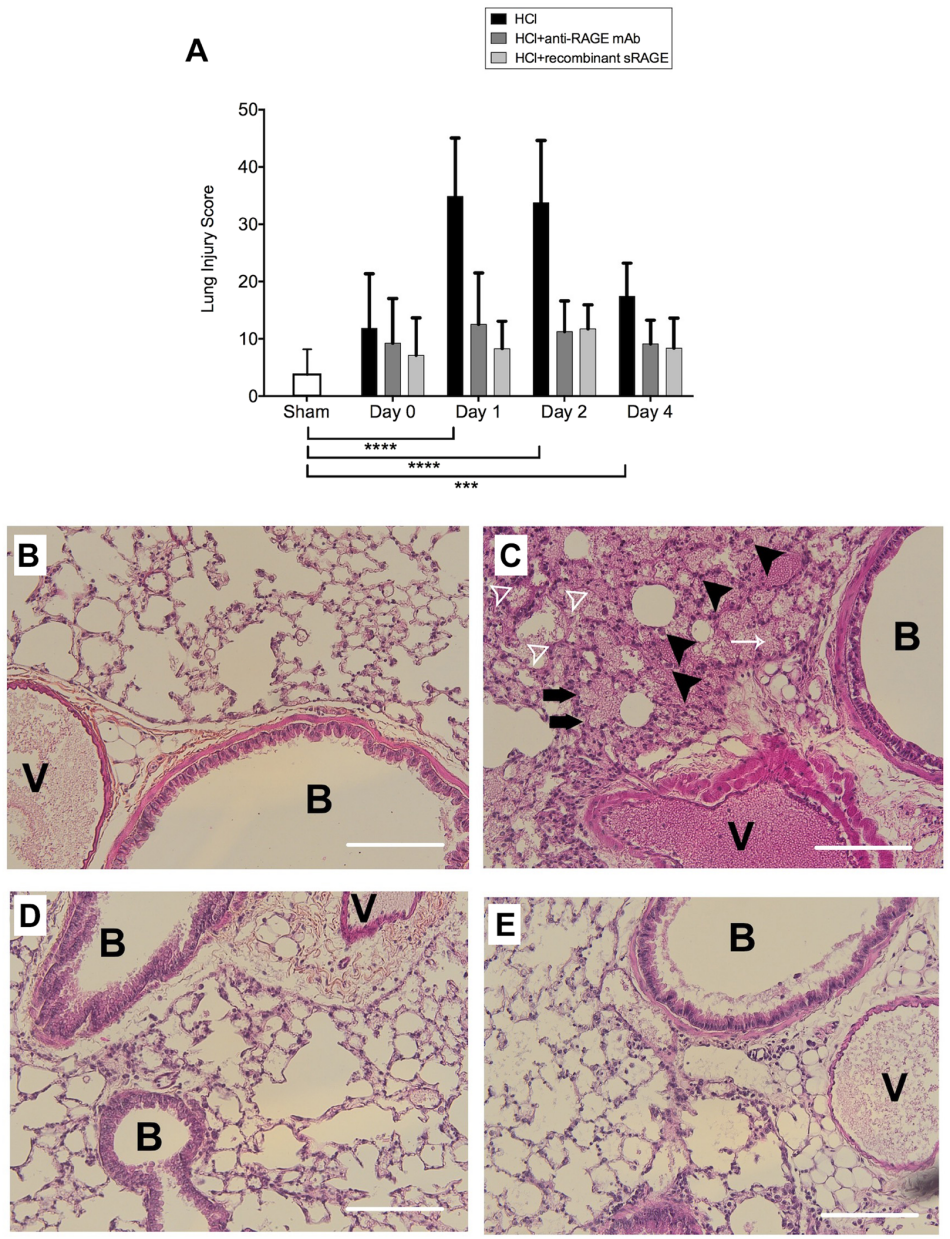
Histological features of lung injury. **(A)** On days 1, 2 and 4, lung injury scores were higher in acid-injured mice (HCl group), compared to sham controls (Sham group)(n = 4–6 for each time-point). In contrast, lung injury scores were lower in mice treated with anti-RAGE mAb (HCl + anti-RAGE mAb group) or sRAGE (HCl + recombinant sRAGE group) than in untreated acid-injured mice (HCl group) at all time-points, and similar to those in sham animals. Lung injury was assessed on a scale of 0–2 for each of the following criteria: i) neutrophils in the alveolar space, ii) neutrophils in the interstitial space, iii) number of hyaline membranes, iv) amount of proteinous debris, and v) extent of alveolar septal thickening. The final injury score was derived from the following calculation: Score = [20x(i) + 14x(ii) + 7x(iii) + 7x(iv) + 2x(v)]/(number of fields × 100). As no difference was observed between sham animals at all time points; the results from sham mice were mixed for analyses (left bar of the X-axis). Values are reported as means ± standard deviations. ***P < 10^−3^; ****P < 10^−4^ versus sham controls. **(B**–**E)** Representative hematoxylin and eosin stained sections of lung tissue, on day 2 after acid injury. **(B)** Sham controls (Sham group), **(C)** acid-injured animals (HCl group) and **(D)** acid-injured animals treated with anti-RAGE monoclonal antibody (HCl + anti-RAGE mAb group), **(E)** acid-injured animals treated with sRAGE (HCl + recombinant sRAGE group). There was greater cellularity consisting mainly of neutrophils (black arrowheads) on day 2 after injury, with more areas of atelectasis and increased alveolar disruption, hyaline membranes (white arrowheads), proteinous debris, haemorrhage (white arrow) and the thickening of the alveolar wall (black arrows). *B: Bronchus lumen, V: Vessel*. Original magnification × 20. Scale bars 50 μm.

Lung injury scores were significantly increased on days 1, 2 and 4 in acid-injured mice as compared to sham mice and injured mice on day 0. In injured mice to whom anti-RAGE mAb or sRAGE was administered, lung injury scores were lower than those in untreated acid-injured mice at all time-points, and similar to those in sham animals (Fig. 7A). Compared to sham mice (Fig. 7B), there were disrupted alveoli, presence of fluid and hemorrhage within the alveolar space, alveolar wall thickening and neutrophilic and mononuclear infiltrate in acid-induced mice on day 2 (Fig. 7C). Acid-induced mice treated with anti-RAGE mAb (Fig. 7D) or sRAGE (Fig. 7E) had less intense neutrophilic infiltration, alveolar oedema and disruption than other untreated, acid-injured mice. Similar findings were overall observed over time (Supplement, Fig. S2).

### Lung expression of alveolar epithelial channels

**Figure 8:**
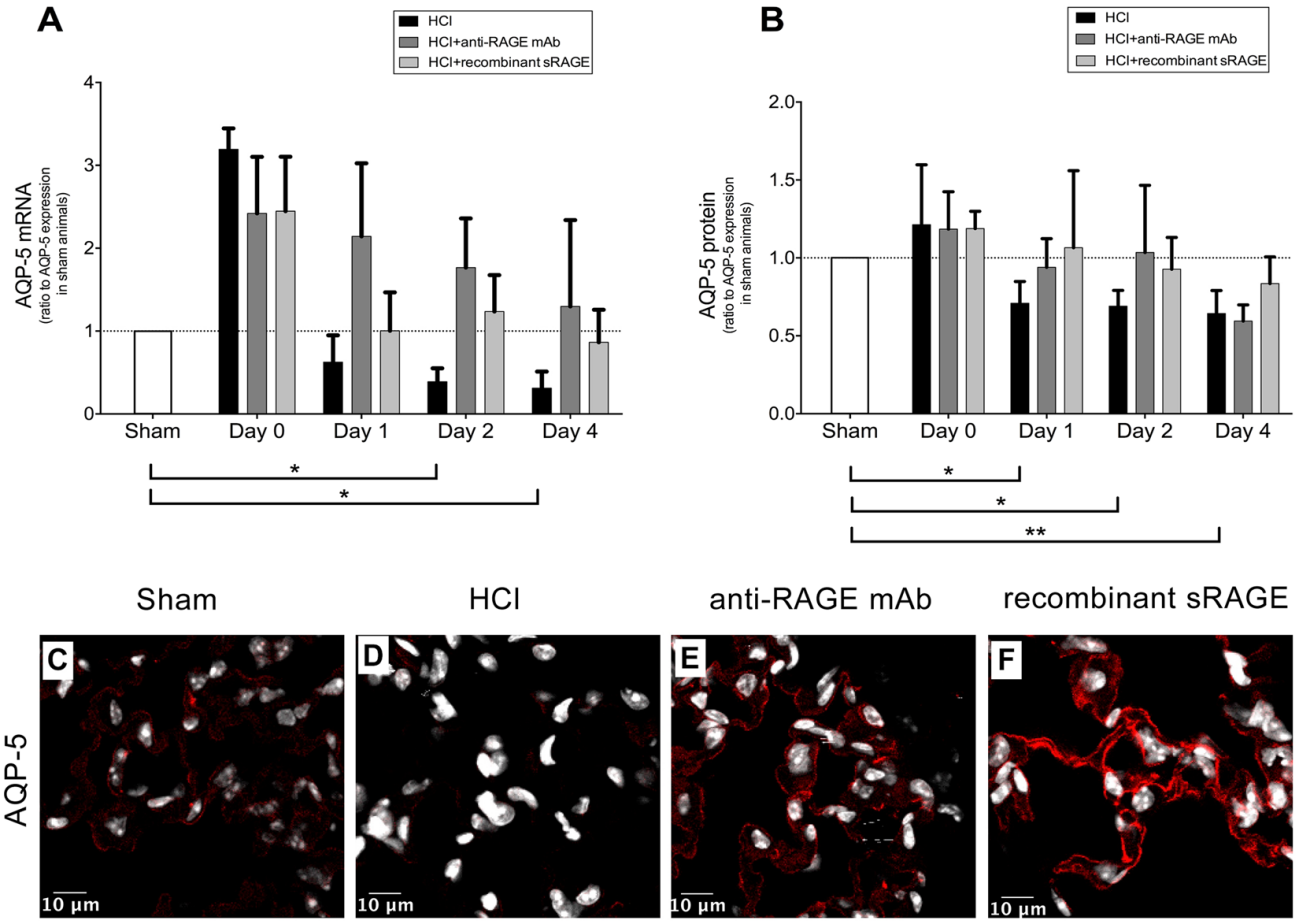
Treatment with both anti-RAGE monoclonal antibody (mAb) and recombinant sRAGE restore Aquaporin (AQP)-5 expression in mouse alveolar epithelial cells. Levels of AQP-5 (**A**) mRNA and (**B**) protein in acid-injured mice (HCl group), acid-injured mice treated with anti-RAGE monoclonal antibody (HCl + anti-RAGE mAb group) or with recombinant sRAGE (HCl + sRAGE group), and in uninjured, untreated mice (Sham group)(n = 4–6 for each time-point). Levels of AQP-5 proteins were measured in duplicate *via* ELISA. Threshold levels of mRNA expression (∆∆Ct) were normalised to housekeeping genes. Protein and mRNA expression levels are expressed as ratios to those in sham animals. As no difference was observed between sham animals at all time points; the results from sham mice were mixed for analyses (left bar of the X-axis). Values are reported as means ± standard deviations. *P < 0.05; **P < 10^−2^ versus sham controls RAGE inhibition restores alveolar membrane expression of AQP-5 in acid-injured mice. Representative photographs of immunohistochemistry on mouse lungs probed at day 1 for **(C**–**F)** AQP-5 protein. Nuclei were labeled with 4′,6-diamidino-2-phenylindole (DAPI). Control slices stained without primary antibodies were always negative (Supplement, Fig. S5). Original magnification ×40. Scale bar = 10 µm.

The expression of AQP-5 in the lung was down-regulated from day 1 to day 4 after injury, and anti-RAGE therapy restored the protein and mRNA expressions of AQP-5 in the lung (Fig. 8A–B). In contrast, acid injury was associated with upregulated lung mRNA expression of *α*1-ENaC and *α*1-Na,K-ATPase on days 1–2, compared to sham animals, whereas lung *α*1-ENaC and *α*1-Na,K-ATPase proteins were decreased from day 1 to day 4 after injury. RAGE inhibition limited the rise in lung *α*1-ENaC mRNA at days 1–4, but had no effect on *α*1-ENaC protein levels. No obvious changes were observed in lung *α*1-Na,K-ATPase protein and mRNA levels in mice treated with anti-RAGE mAb or sRAGE (Supplement, Fig. S3A–D).

### Localisation of epithelial channels in alveolar cells

Immunostaining revealed the disruption in both AQP-5 (Fig. 8C–D) and *α*1-ENaC (Supplement, Fig. S4A–B) proteins in the alveolar cell membranes of injured lungs, compared to those from sham controls. *α*1-Na,K-ATPase was stained in nuclei, rather than in membranes, and acid injury decreased fluorescence (Supplement, Fig. S4E–F). RAGE inhibition had no clear effect on *α*1-ENaC (Supplement, Fig. S4C–D) and *α*1-Na,K-ATPase (Supplement, Fig. S4G–H) staining, whereas treatment with either anti-RAGE mAb or sRAGE restored the membrane expression of AQP-5 (Fig. 8, E–F).

## Discussion

Our main goal was to determine the impact of a RAGE inhibition strategy in a model of direct epithelial lung injury^13, 26^. In this study, we demonstrated that both sRAGE and anti-RAGE antibody had similar therapeutic effects in experimental ARDS, with improved arterial oxygenation, restored AFC, and attenuated histological lung injury, alveolar capillary permeability and inflammation. Notably, anti-RAGE therapy possibly decreased AT-1 cell injury, as suggested by the recovery of AQP-5 protein and mRNA expression in mouse lungs, compared to untreated, acid-injured controls.

Because there is significant upregulation of RAGE expression during ARDS^6, 13, 27^, decreasing RAGE activation could be a therapeutic target in lung injury^28^. To date, the use of both sRAGE and anti-RAGE antibodies has been associated with beneficial effects on lung injury, among other diseases. In a sepsis model, the absence of RAGE was associated with improved survival after cecal ligation and puncture (CLP)^25^. In this study, an anti-RAGE mAb decreased mortality, even when given 24 h after CLP. Furthermore, a humanised anti-RAGE mAb protected mice from pneumonia-induced mortality^29^, thus suggesting the notion that direct RAGE antagonism could be a promising therapeutic target in lung injury^28^.

Another approach to neutralising RAGE-ligand interaction is the administration of C-terminal truncated RAGE such as sRAGE, i.e. a decoy receptor that retains the capacity to bind to ligands, which would otherwise interact with full-length mRAGE^7^. The balance between RAGE isoforms has been described as an important regulator of RAGE activation, under both physiological and pathological conditions^30^. Generally, mRAGE may rather promote disease pathogenesis and injury by activating the NF-κB pathway, while sRAGE may rather be protective by preventing or reversing mRAGE signaling in diseases^19, 31^. Because recombinant sRAGE has an elimination half-life of 49 h after intraperitoneal injection into normal rats^32^ and because BAL and plasma sRAGE remained elevated over time in our study, we hypothesise that the effects of sRAGE could be sustained enough to explain its beneficial effects in our model on days 1–2. We further hypothesise that lung RAGE mRNA expression resulting from acid-induced activation of RAGE pathway may, in turn, increase circulating sRAGE through various mechanisms in order to counterbalance self-perpetuating activation of mRAGE by its ligands^33^. Further blockade of RAGE with anti-RAGE mAb or sRAGE in our study may have restrained mRAGE-ligand interactions, thus possibly contributing to restored AFC and decreased levels of proinflammatory cytokines. Such anti-inflammatory effects of anti-RAGE therapy could explain improved features of lung injury in acid-injured treated mice. Indeed, in a rabbit model of direct lung injury, Folkesson *et al*. showed that a treatment with an anti-IL-8 mAb prevented neutrophil influx into the air spaces of the lung^34^. IL-8 neutralization led to both an increase in oxygenation and a decrease in extravascular lung water, thus strengthening the important implications of interleukin modulation for the treatment of acute lung injury after inhalation.

In addition to the less pro-inflammatory state induced by anti-RAGE therapies, both sRAGE and anti-RAGE mAb prevented the upregulation of RAGE mRNA in injured mice, thus providing a potential mechanistic link between peripheral RAGE inhibition and injury attenuation in lungs. Conversely, a restoration of both RAGE and AQP-5 protein expression was observed in lungs of treated, acid-injured mice, thus suggesting a potential restoration of AT-1 epithelial cell integrity. Taken together, these results emphasize the complex roles of RAGE in lung injury, and further strengthen the hypothesis of dichotomous roles of RAGE during lung injury, as both a biomarker of lung epithelial injury and an amplifier of inflammation.

Interestingly, anti-RAGE therapies were associated with full restoration of AQP-5 mRNA and protein expressions on days 1–2 after injury. However, the role of AQP-5 in AFC remains controversial during lung injury. Indeed, although this role might be more important than previously thought^35, 36^, it has been reported that AQP-5 deletion had no effect on lung fluid accumulation or active fluid absorption^37^. In our study, the restored expression of AQP-5 in mice treated with anti-RAGE therapy could, at least, reflect decreased AT-1 epithelial cell injury. In addition, one could hypothesise that beneficial effects of anti-RAGE therapies could be related to improved airway surface liquid properties of the lungs. Indeed, it has been reported that lung injury in AQP-5 null mice after *Pseudomonas Aeruginosa* challenge could be due to lung surfactant changes, in which AQP-5 deficiency leads to reduced mucin production by the lung and declined activation of mitogen-activated protein kinases and nuclear factor-κB^38^.

Our findings also provide new insights into time-dependent *in vivo* perturbations of AFC after acid injury, which have already been described with regard to α-ENaC expression after LPS^39^. The mRNA expression of α1-ENaC and α1-Na,K-ATPase in the acid-induced lungs was upregulated in our model, without any concurrent changes in protein expression. Beyond the recent demonstration that RAGE activation stimulates the assembly and activation of ENaC through protein kinase C (PKC)-gp91^phox^ signaling^18^, more work is needed to better characterize specific effects of RAGE activation by its various ligands on intracellular pathways, and how these RAGE-dependent effects precisely regulate the mechanisms of fluid balance. Indeed, it is speculated that each RAGE ligand has distinct effects upon RAGE’s activation, through the engagement of specific intracellular pathways^30^.

Current pharmacotherapy measures against ARDS have been limited in terms of improving outcomes^2^. Despite our promising results, the actual benefit of RAGE inhibition in humans with ARDS is still unknown. Some successfully options such as sRAGE decoys have already been reported^40^, but specific strategies targeting a particular signaling pathway downstream of RAGE should be further explored under cell-type specific conditions. A complete understanding of the relationships between RAGE, epithelial integrity and mechanisms of AFC will be beneficial in formulating new potential therapeutic approaches. As of now, the modification of the RAGE-ligand activation pathway seems to be a promising target but interventions designed to modulate immune responses have historically been disappointing when assessed in clinical trials^41^. Inhibition of RAGE pathway may have a greater chance of improving clinical outcome as compared to previous candidates, especially because RAGE inhibition remains efficacious even when given hours after experimental injury in many studies^21, 25^. To date, a RAGE inhibitor has already been investigated in a clinical setting of Alzheimer’s dementia, with inconclusive results^42^. However, there are reasons to be cautious when considering the potential therapeutic application of RAGE blockade in diseases such as ARDS, especially because an intact RAGE axis may be necessary for inflammatory response. Although the beneficial effects found in preclinical studies may not be reflected in clinical studies, measuring circulating sRAGE could be used as a tool to identify subjects who may benefit more greatly from RAGE inhibition.

Our study has some potential limitations. First, one of the factors limiting the clinical translation of preclinical findings is the limitations of *in vivo* disease models, but animal experiments remain essential to understanding the fundamental mechanisms underlying the onset of diseases and to discovering improved methods to preventing, diagnosing and treating them. Second, we limited our evaluation at day 4 after injury and did not assess later time-points^26^. Whether RAGE inhibition could impact later endpoints, including the development of lung fibrosis, was not investigated here^26^. Third, we acknowledge analytical interferences between ELISA measurements of sRAGE and the administration of recombinant sRAGE or anti-RAGE mAb which requires additional caution in the interpretation of sRAGE levels in treated animals. Fourth, although treated mice had no obvious phenotypic change during our experiments^13, 26^, both organ- and ligand-specific effects of RAGE inhibition were out of the scope of this study, thus prompting further investigations^21, 43–45^. Fifth, the mechanisms through which systemic anti-RAGE therapy may alleviate acid-induced lung injury are largely unknown to date. Although the hypothesis that it could interact with circulating RAGE ligands has already been suggested, our study is unable to provide definite answers. Unfortunately, we did not measure RAGE ligands neither in the plasma or in the BAL in this study, although such data would be helpful to better understand the roles and kinetics of RAGE ligands in lung injury and repair. However, it should also be acknowledged that many RAGE ligands can interact with other receptors, and that RAGE can interact with many other ligands as well^30^. It has previously been found that patients with ARDS had higher sRAGE, HMGB1 and S100A12 levels than patients without ARDS. Inversely, esRAGE and AGEs levels were lower in patients with ARDS than in those without the syndrome^27^. These findings support a role for increased HMGB1 and S100A12 levels in lung injury, although further mechanistic studies are warranted. Sixth, the translation of possible beneficial effects of anti-RAGE therapies into the clinical setting of lung injury needs extreme caution, given that no study has been performed to date in the setting of sepsis, the most frequent cause of ARDS. Finally, correlation between RAGE inhibition and restored AFC does not imply causation, and more mechanistic studies are now needed to describe mechanisms through which RAGE might impact AFC and AQP-5 expression, and the roles of RAGE pathway on other apical sodium channels^15^ or on other factors that can influence the resolution of alveolar oedema (e.g., cell proliferation and differentiation, intercellular crosstalks and tight junctions).

Our study also has important strengths. First, a strategy of RAGE inhibition has never been reported in a translational mouse model of direct epithelial injury so far. Second, no study has compared two modalities of RAGE inhibition in experimental ARDS to date, and our findings support similar beneficial effects of sRAGE and anti-RAGE mAb on lung injury and AFC. Because anti-RAGE mAb was administered before acid instillation and recombinant sRAGE after injury, targeting RAGE could have a protective role in both the preconditioning and postconditioning settings. Finally, this model of both the onset of lung injury and its resolution may be particularly relevant to studies of AFC, a phenomenon that is often impaired in ARDS, a life-threatening syndrome with limited effective therapy.

In conclusion, the use of both recombinant sRAGE and anti-RAGE mAb alleviated lung injury, improved arterial oxygenation, AFC, and restored lung AQP-5 expression in a translational mouse model of ARDS. Our findings should prompt further mechanistic studies of the pathways from RAGE activation to AFC and regulation of alveolar epithelial channels.

## Materials and Methods

### Animal model

Mice were maintained and procedures were performed with the approval of the Auvergne Regional Ethics Committee (CEMEA Auvergne) in the animal facility of the School of Medicine – University of Clermont-Ferrand (approval number CE 67–12). All experiments were performed in accordance with relevant guidelines and regulations. Male C57BL/6JRj mice (Janvier Labs, Saint-Berthevin, France), aged 10–12 weeks and weighing 25–30 g, were anesthetised *via* an intraperitoneal injection of xylazine (10 mg/kg) and ketamine (100 mg/kg), and given an intraperitoneal fluid bolus of 10 µL/g 0.9% isotonic saline as pre-emptive fluid resuscitation. The mice were suspended vertically from their incisors on a custom-made mount for orotracheal instillation, as described previously^13, 26^. A fine catheter was guided 1 cm below the vocal cords, and 75 µL of an iso-osmolar (to mouse plasma, i.e. 322 mOsm/L) solution of 0.1 M hydrochloric acid (pH 1.0) were instilled^13, 26^. For the next 4 h, during which time animals exhibited significant respiratory distress, the mice were kept in a transparent recovery box under humidified supplemental oxygen (inspiratory oxygen fraction (FiO_2_) reduced gradually from 1.0 to 0.21). During this period, the animals were carefully monitored, and their body temperature was maintained using external heat sources, after which they were transferred to individually ventilated cages with air and free access to food and water. For technical reasons, investigators who performed animal experiments and who collected samples were not blinded to treatment groups. Nevertheless, technicians who analysed biological samples and the statistician who performed statistical analyses were blinded.

To examine the effects of RAGE inhibition, lung-injured mice were divided into three groups. Acid-injured mice (HCl group) received an intratracheal instillation of HCl (75 μl/mouse). The mAb group was injected intravenously with anti-RAGE monoclonal antibody (mAb) (15 mg/kg, R&D Systems, Minneapolis, MN or 1% autologous mouse serum control) 30 minutes prior the HCl instillation^25^, and the soluble RAGE (sRAGE) group was administered recombinant mouse sRAGE (100 μg/mouse, R&D Systems, Minneapolis, MN or 100 μg/mouse of saline as control) intraperitoneally 1 h after the HCl instillation^21^.

### Physiological measurements

The criteria for experimental ARDS were evaluated as recommended by the *American Thoracic Society* ^46^ at baseline (day 0) in injured and sham animals, and at specified time-points (days 1, 2 and 4) after acid instillation in injured mice^13, 26, 46^. After an initial lung recruitment manoeuvre (30 cm H_2_O for 5 s), the animals were ventilated for 30 min (tidal volume 8–9 mL/kg, positive end-expiratory pressure 6 cm H_2_O, respiratory rate 160 breaths/min and FiO_2_ 1) to standardise the volume history of the lungs. At the end of ventilation, blood gases were measured, and the mice were sacrificed *via* anaesthetic overdose with intraperitoneal pentobarbital (150 mg/kg). Acid-injured animals were compared with injured mice treated with anti-RAGE mAb or recombinant sRAGE, and with otherwise sham mice, receiving only saline tracheal instillation, surgical preparation and 30-min of ventilation. All mice received 10 mg/kg of human serum albumin (HSA) dissolved in 100 μL of saline intravenously, 1 h before euthanasia, for the measurement of the lung permeability index, presented as a percentage. This permeability index was defined as the ratio of HSA in the BAL fluid to that in the plasma collected at the end of the experiments^22^. The HSA concentration was measured in duplicate by enzyme linked immunosorbent assay (ELISA) using a human albumin ELISA Kit (R&D Systems, Minneapolis, MN). The lower limit of detection was 5 ng/mL.

### Alveolar fluid clearance

AFC was evaluated at baseline (day 0) and on days 1, 2 and 4 after acid instillation, in injured and sham animals, using a modification of previous *in situ* models^13, 26, 46^. A bovine serum albumin (BSA) solution was instilled into the trachea; after 30 min of ventilation (tidal volume = 8 mL/kg, positive end-expiratory pressure = 6 cm H_2_O, respiratory rate = 160 breaths/min and FiO_2_ = 1), proteins were measured in the instilled fluid to calculate net AFC rate: percent AFC over 30 min = 100 × [1 − (initial/final total protein)]. In mice, the initial protein concentration was estimated by the protein concentration of the BSA instillate.

### Assessment of inflammation and lung epithelial injury

In separate animals, BAL was performed with 750 µL of saline as described previously^13, 26^ and systemic blood was drawn from a cardiac puncture; the samples were centrifuged at 240 × g. The protein levels in the BAL fluid were quantified in duplicate with a colorimetric method (Pierce Biotechnology, Rockwood, IL). The BAL and plasma levels of interleukin (IL)-6, tumor necrosis factor (TNF)-α, IL-17, keratinocyte-derived chemokine (KC) and macrophage inflammatory protein (MIP)-2 were determined in duplicate using the Bio-Plex 200 System, which is based on Luminex xMAP Technology (Bio-Rad, Hercules, CA, USA). In the present study, we screened BAL and plasma samples using the Mouse Cytokine 4-plex panel and MIP-2 SET (Bio-Rad, Marnes-la-Coquette, France). Cytokine levels were normalised to total protein levels. BAL and plasma levels of sRAGE were measured in duplicate using ELISA (R&D Systems, Minneapolis, MN). BAL cell counts were obtained using a hemocytometer.

### Lung mRNA and protein expression

The animals were sacrificed after the ventilation period and AFC measurements, and subjected to lung sampling for the assessment of protein and mRNA expression levels. A Mem-PER Plus Membrane Protein Extraction Kit (Thermo Scientific, Weltham, MA) was used to extract membrane proteins of the right lung tissues of mice in each treatment group, following the manufacturer’s instructions. Briefly, 50 mg left lung tissue were washed with cell wash solution, cut to pieces and homogenised in permeabilization buffer to an even suspension. The cells were scraped off, resuspended in Hites medium and centrifuged at 300 × g for 5 minutes. The cell pellet was washed with 3 mL of cell wash solution and centrifuged at 300 × g for 5 min. After the complete removal of the supernatant containing the cytosolic extract, the cells were resuspended in wash solution and centrifuged at 300 × g for 5 min. Again, the supernatant was discarded and permeabilization buffer was added to cell pellet. Then the homogeneous tissue and cell suspension in permeabilization buffer was obtained and incubated at 4 °C with mixing for 20 min. Again, the pellet was centrifuged at 16,000 × g for 15 minutes and the supernatant was discarded. The pellet was resuspended in solution buffer and incubated at 4 °C with mixing for 40 min. The suspension was centrifuged at 16,000 × g for 15 minutes at 4 °C. Finally, the membrane protein contained in supernatant was obtained and further quantified in duplicate using mouse RAGE quantikine (R&D Systems, Minneapolis, MN), AQP-5 (MyBiosource, San Diego, CA), α1-ENaC and α1-Na,K-ATPase (Antibodies-online Inc, Atlanta, GA) ELISA kits.

In parallel, total RNA was isolated from the left lung with an RNA extraction kit (RNeasy® Mini Kit, Qiagen, Valencia, CA). The gene expression levels of RAGE (mRNA Refseq NM_007425), α1-ENaC (NM_011324), α1-Na,K-ATPase (NM_144900), and AQP-5 (NM_009701) were assessed using semi-quantitative real-time polymerase chain reaction (PCR)(mouse RT² Profiler™ PCR Array, Qiagen, Valencia, CA). Threshold levels of mRNA expression (∆∆Ct) were normalised to housekeeping genes, and the values represent the mean of triplicate samples ±standard deviation (SD). Data are representative of three independent observations. Housekeeping genes included the following: Guanine nucleotide binding protein (G protein), beta polypeptide 2-like 1; Ribosomal protein, large, P0; beta-2-microglobulin; TATA box binding protein; phosphoglycerate kinase 1.

### Histological examination and immunofluorescence

At each time-point, acid-injured and untreated animals were sacrificed and their right lungs were removed, fixed with alcoholic acetified formalin and embedded in paraffin. The histological analyses were carried out by one independent expert, blinded to the treatment. Slices at 10-μm thickness were subsequently stained with hematoxylin and eosin (Sigma-Aldrich Ltd). A standardised histology injury score was derived from the following calculation: score = [20x(i) + 14x(ii) + 7x(iii) + 7x(iv) + 2x(v)]/(number of fields × 100) (supplementary, table S13)^46^. In separate animals and after sacrifice, both of their lungs were fixed *in situ* through the trachea with 4% paraformaldehyde at a pressure of 1.96 kPa (20 cm H_2_O). All specimens were paraffin embedded, cut to 4 μm thickness and then deparaffinised in Histoclear. The buffer solutions used for heat-induced epitope retrieval were Tris-EDTA buffer solution (10 mM Tris Base, 1 mM EDTA Solution, pH 9.0) for AQP-5, ENaC and Na,K-ATPase. Slices were then washed and blocked for 1 h at room temperature in a humidified incubator with 1% BSA or 5% NGS. The following primary antibodies were used overnight: rabbit polyclonal anti-AQP-5 antibody (1:400, Merck Millipore), rabbit polyclonal anti-α1-ENaC antibody (1:1000, Abcam) and rabbit polyclonal anti-α1-Na,K-ATPase antibody (1:500, Abcam). Goat anti-rabbit and donkey anti-goat secondary antibodies coupled with Alexa Fluor^®^ 555-F (Molecular Probes) dye (Thermo Fisher Scientific) were used at a dilution of 1:1000. Control slices without primary antibodies were used as a negative control for the nonspecific binding of the secondary antibody (Fig. S5). The sections were then incubated for 5 min with Hoechst at 1 µg·ml^-1^ (Sigma-Aldrich), rinsed, mounted in PBS-glycerol, and photographed under a microscope (Confocal Fluorescence Imaging Microscope, Leica TCS-SP5) at 40 × magnification. Image processing was performed by Image J software.

### Statistical analysis

Categorical data were expressed as numbers and percentages, and quantitative data were expressed as means ± SDs or medians and interquartile ranges (IQRs) according to statistical distribution. For studies of protein and mRNA expression levels, data are presented as ratios to the expression levels in control cell conditions or in sham animals. Statistical analyses were carried out *via* Kruskal-Wallis tests with Bonferroni tests for pairwise comparisons between each time-point and sham controls (represented as day 0), or between various cell culture conditions. A limited number of animals were used for baseline comparisons (n = 3–4), and four to six animals were used in each group on days 1, 2 and 4 in order to detect a difference of 5% per 30 min (SD = 2.5) in net AFC rate on day 1 or day 2 between acid-injured animals and those treated with either sRAGE or anti-RAGe mAb, when considering alpha and beta risks of 5% (bilateral) and 10%, respectively. A statistical power of 90% was considered sufficient to allow multiple comparisons between groups. Analyses were performed using Prism 6 (Graphpad Software, La Jolla, CA). A P < 0.05 (two-sided) was considered significant.

## Electronic supplementary material

Supplementary Informations
