# Supplementary material for: RAGE inhibition reduces acute lung injury in mice
Source: Sci Rep. 2017 Aug 3;7:7208. doi: 10.1038/s41598-017-07638-2 (PMC5543147; doi:10.1038/s41598-017-07638-2)
Supplement: Supplementary file 1 — Supplementary Informations [file 41598_2017_7638_MOESM1_ESM.doc]

**Supplementary Information**

**RAGE inhibition reduces acute lung injury in mice**

Raiko Blondonnet*, Jules Audard, Corinne Belville, Gael Clairefond, Jean Lutz, Damien Bouvier, Laurence Roszyk, Christelle Gross, Marilyne Lavergne, Marianne Fournet, Loic Blanchon, Caroline Vachias, Christelle Damon-Soubeyrand, Vincent Sapin, Jean-Michel Constantin & Matthieu Jabaudon

**TABLE**

| Parameter | 0 | 1 | 2 |
| --- | --- | --- | --- |
| i. Neutrophils in the alveolar space | None | 1-5 | >5 |
| ii. Neutrophils in the interstitial space | None | 1-5 | >5 |
| iii. Hyaline membranes | None | 1 | >1 |
| iv. Proteinous debris filling the airspaces | None | 1 | >1 |
| v. Alveolar septal thickening | None | 2x-4x | >4x |

**Table S1**.  **Lung injury scoring system** (adapted from Matute-bello et al [[5]](https://paperpile.com/c/Ky7RC4/ZWnGs)).

**FIGURES**

**
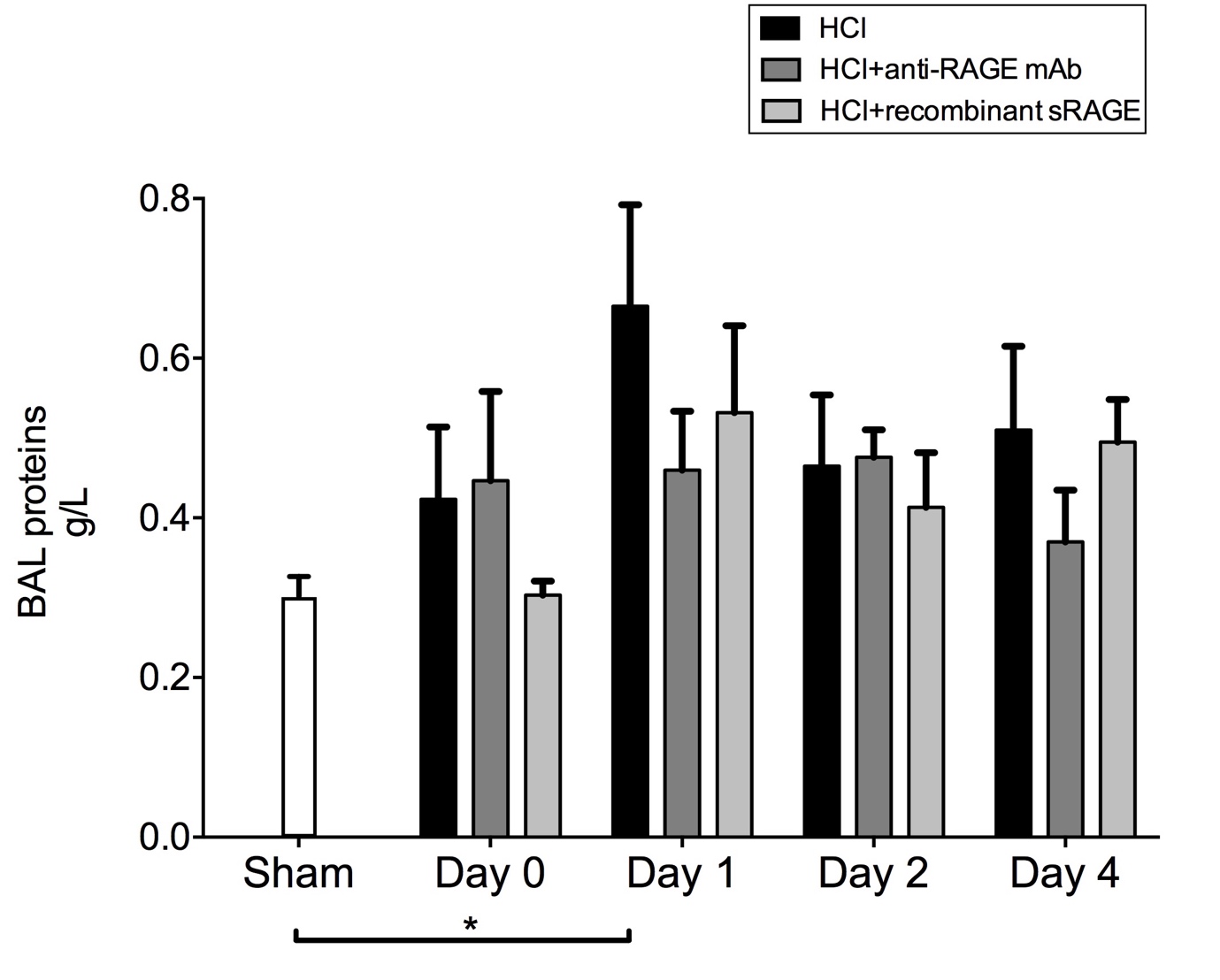
**

**Figure S1. RAGE inhibition decreases total bronchoalveolar lavage (BAL) protein concentration**. The protein levels in the BAL fluid were quantified in duplicate with a colorimetric method (Pierce Biotechnology, Rockwood, IL) in uninjured untreated mice (Sham group), acid-injured animals (HCl group) and acid-injured animals treated with sRAGE (HCl+recombinant sRAGE group) or with anti-RAGE monoclonal antibody (HCl+anti-RAGE mAb group)(n=4–6 for each time-point). Values are reported as means ± standard deviations. *: P<10^-2^ versus sham controls.

**
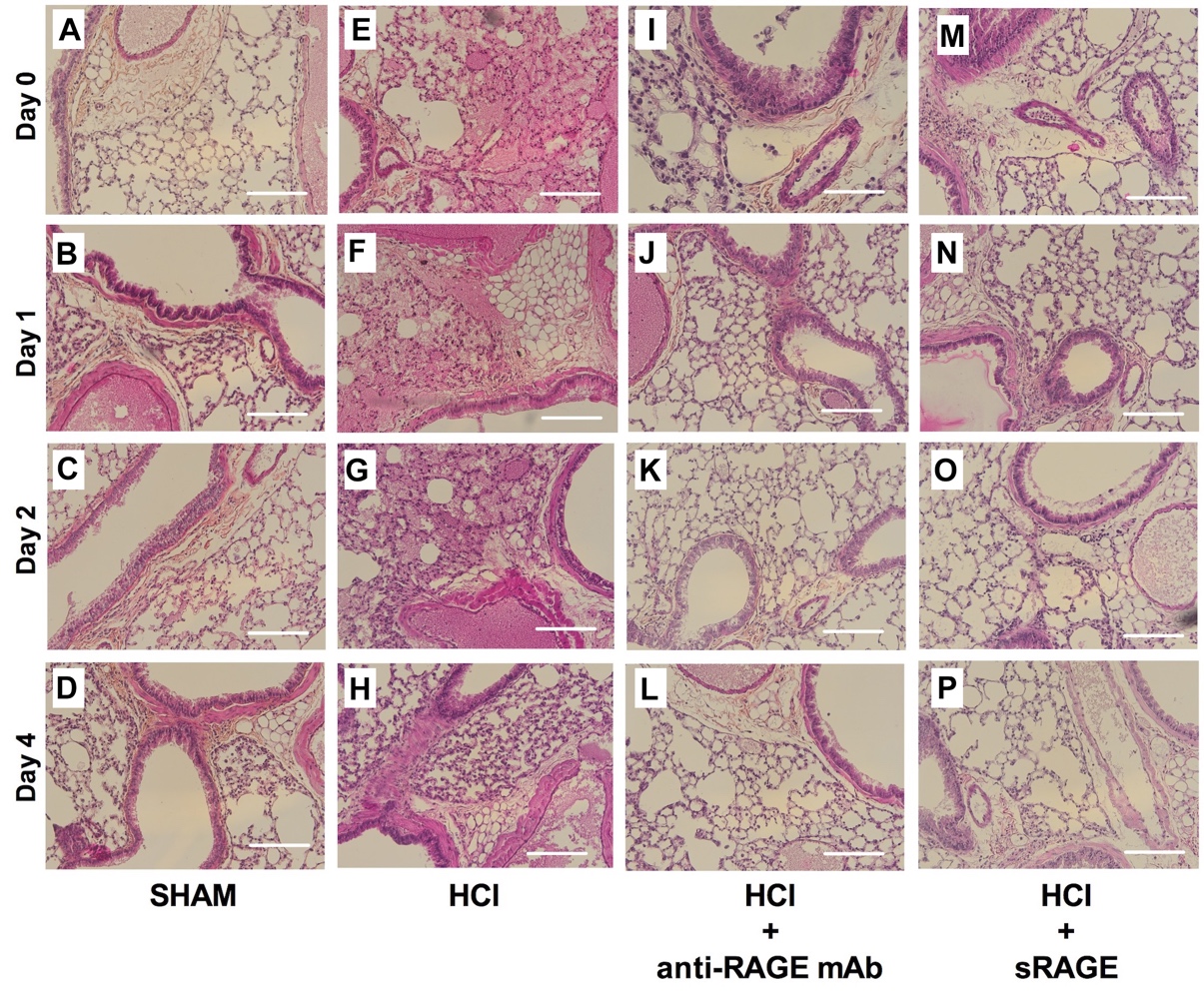
**

**Figure S2.** **Changes over time in histological evidence of tissue injury.** Representative hematoxylin and eosin stained sections in different groups of animals: **(A-D)** uninjured, untreated mice (Sham group), **(E-H)** acid-injured mice (HCl group) **(I-L)** acid-injured mice treated with anti-RAGE mAb (HCl+anti-RAGE mAb group), **(M-P)** acid-injured mice treated with recombinant sRAGE (HCl+sRAGE group). Compared to sham and acid-injured mice treated with sRAGE or anti-RAGE mAb, untreated acid-injured animals had greater cellularity (consisting mainly of neutrophils), wider areas of atelectasis, increased alveolar disruption with hyaline membranes, proteinous debris, hemorrhage and thickening of the alveolar wall at all time-points. Mice treated with either anti-RAGE mAb or sRAGE had increased neutrophil infiltration and proteinous debris into the alveoli at baseline **(I-M)**, compared to sham controls **(A-E)**. At days 1–2, histological features of lung injury were decreased in acid-injured mice treated with either anti-RAGE mAb or sRAGE, compared to untreated acid-injured mice (**K-L and O-P**). Original magnification x20. Scale bars 50 μm.

**
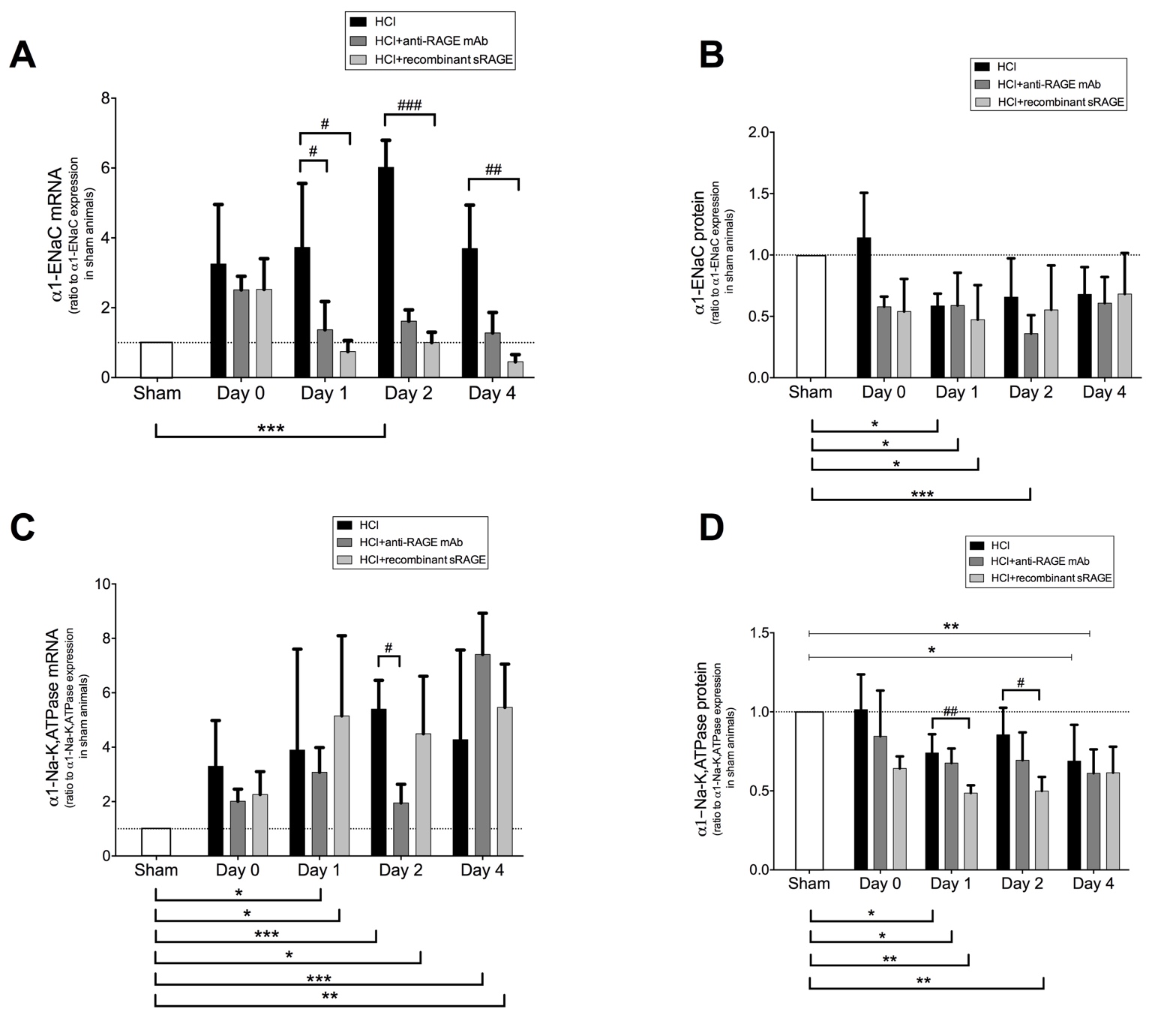
**

**Figure S3.**  **Effects of treatment with both anti-RAGE monoclonal antibody (mAb) and recombinant sRAGE on ENaC and Na,K-ATPase expression in mouse alveolar epithelial cells.** Levels of ɑ1-ENaC (A) mRNA and (B) protein and ɑ1-Na,K-ATPase (C) mRNA and (D) protein in acid-injured mice (HCl group), acid-injured mice treated with anti-RAGE monoclonal antibody (HCl+anti-RAGE mAb group) or with recombinant sRAGE (HCl+sRAGE group), and in uninjured, untreated mice (Sham group)(n=4–6 for each time-point). Levels of ɑ1-ENaC and ɑ1-Na,K-ATPase proteins were measured in duplicate *via* ELISA. Threshold levels of mRNA expression (∆∆Ct) were normalised to housekeeping genes. Protein and mRNA expression levels are expressed as ratios to those in sham animals. Values are reported as means ± standard deviations. *: P<0.05; **: P<10^-2^; ***: P<10^-3^ versus sham controls; #: P<0.05; ##: P<10^-2^; ###: P<10^-3^ versus acid-injured animals.


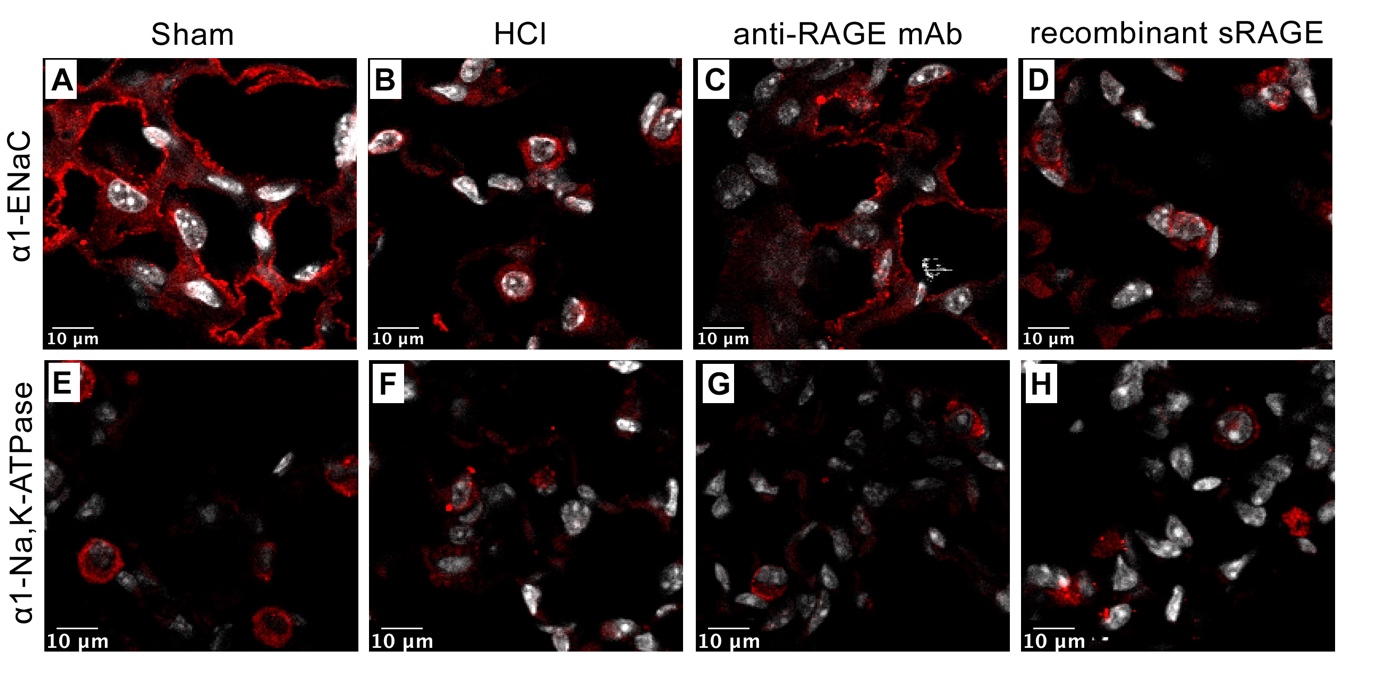


**Figure S4**. **RAGE inhibition has no clear effect on alveolar membrane expression of ENaC and Na,K-ATPase in acid-injured mice**. Representative photographs of immunohistochemistry on mouse lungs probed at day 1 for (A-D) ɑ1-ENaC and (E-H) ɑ1-Na,K-ATPase. Nuclei were labeled with 4’,6-diamidino-2-phenylindole (DAPI). Control slices stained without primary antibodies were always negative (Supplemental Fig. S4). Original magnification x40. Scale bar=10µm.

**
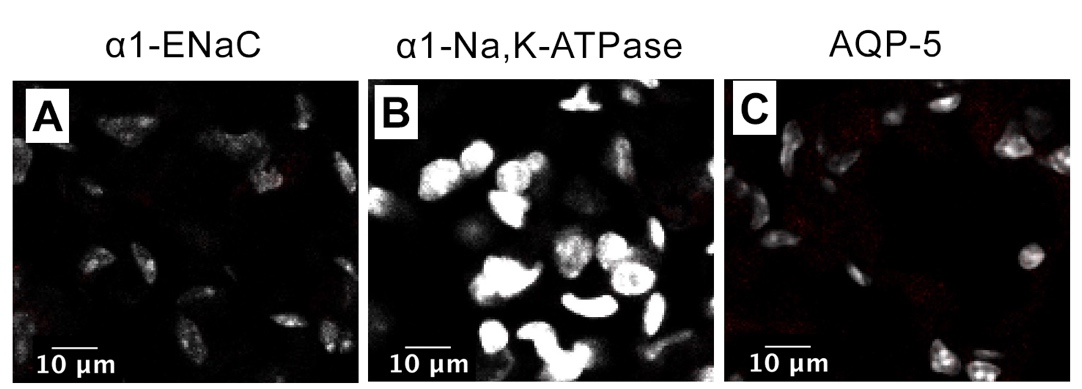
**

**Figure S5**. **Negative controls for immunohistochemistry analysis** of (A) α1-ENaC, (B) ɑ1-Na,K-ATPase, and (C) AQP-5. All control slices stained without primary antibodies showed no immunostaining of AQP-5, α1-ENaC or ɑ1-Na,K-ATPase proteins in mouse lungs. Original magnification x40. Scale bar=10µm.
